# Long-Term Sequelae in Young Convalescent COVID-19 Patients

**DOI:** 10.1155/2022/9613600

**Published:** 2022-04-25

**Authors:** Ayumi Nakamura, Thomas J. Farrer, Andy Liu

**Affiliations:** ^1^Department of Psychiatry and Behavioral Sciences, Duke University Medical Center, Durham, NC, USA; ^2^Department of Neurology, Duke University Medical Center, Durham, NC, USA

## Abstract

As of March 2022, over 78 million cases of COVID-19 and 900,000 deaths have been reported in the United States. The consequences in the acute phase due to the SARS-COV-2 infection are well defined. Beyond the direct effects of severe acute respiratory syndrome coronavirus-2 (SARS-CoV-2) involving the lung parenchyma, the post-viral complications within the central nervous system are still largely unknown, and a comprehensive evaluation regarding the long-term neuropsychological sequelae from this disease is not well characterized. An increasing number of patients previously diagnosed with COVID-19 have now presented with ongoing symptoms of inattention, executive function, and memory difficulties. These symptoms are collectively and commonly known by the public as ‘brain fog', with many expressing concerns over their inability to engage in the workplace due to these symptoms. Here, we describe three patients who were seen in the Memory Disorders Clinic at Duke University to characterize the long-term neuropsychological symptoms, neuropsychological test results and brain MRI findings after infection with SARS-CoV-2 in a cohort of patients under the age of 60.

## 1. Introduction

In December 2019, a novel coronavirus known as Severe Acute Respiratory Syndrome coronavirus 2 (SARS-CoV-2) was identified in Wuhan, located in the Hubei Province of China. On January 31, 2020, the World Health Organization (WHO) issued a global health emergency, and the disease resulting from SARS-CoV-2, coronavirus disease 2019 (COVID-19), was declared a pandemic on March 11, 2020.

SARS-CoV-2, a positive-sense single-stranded RNA virus that primarily manifests with respiratory complications, was isolated from airway epithelial cells in patients [1]. The symptoms of COVID-19 that develop 2–14 days after exposure to SARS-CoV-2 include fever, chills, shortness of breath, fatigue, anosmia, or ageusia, and the course of illness can range from mild to severe illness requiring hospitalization. Characterization of the SARS-CoV-2 viral genome *via* next-generation sequencing led to the identification of the angiotensin-converting enzyme 2 (ACE2) receptor as the receptor that is recognized by SARS-CoV-2 [2]. The SARS-CoV-2 spike protein binds to the ACE2 receptor in airway epithelia and lung parenchyma and uses the receptor as an access point to enter host cells, thereby mediating infection and acute lung injury [3]. In addition to invasion of the respiratory tract, SARS-CoV-2 also has the potential to invade the nervous system [4]. The ACE2 receptor is expressed widely throughout the brain in both neurons and glia in the subfornical organ, paraventricular nucleus, nucleus of the tractus solitarius, and rostral ventrolateral medulla [5]. Consistent with the ability of other coronaviruses to infect brainstem neurons *via* the ACE2 receptor to contribute to dyspnea and respiratory failure in patients, SARS-CoV-2 may act through similar mechanisms [5, 6].

Those who recover from COVID-19 can manifest several extrapulmonary symptoms. Data from previous epidemics and outbreaks with coronaviruses, including SARS-CoV-1 (in 2003) and Middle East Respiratory Syndrome (MERS), have shown a diverse array of neuropsychiatric sequelae [7, 8], though the exact mechanism by which this occurs remains unclear. Recent reports of patients who were diagnosed with COVID-19 have revealed the neuroinvasive potential of SARS-CoV-2. Infection with this coronavirus can result in a myriad of neurological and psychiatric complications post-infection [7]. These complications include an increased incidence of anosmia, ageusia, headaches, cerebrovascular accidents, meningoencephalitis, as well as cognitive dysfunction [7, 9–12] and were recently coined under the collective term of post-acute sequelae of SARS-CoV-2 infection, or PASC. Though the acute manifestations of COVID-19 are well characterized [13–15], the long-term cognitive consequences in long-haulers, who experience a post-viral syndrome weeks or even months after the initial onset of symptoms, are only beginning to be recognized [7, 16, 17]. Here, we report the development of cognitive dysfunction in a series of individuals younger than age 60 who were diagnosed with and subsequently recovered from COVID-19 infection.

## 2. Case Presentation

### 2.1. Case #1

A 19-year-old Caucasian female with a history of multiple concussions, migraine headaches, autonomic dysregulation and unexplained fevers presented to the Memory Disorders Clinic five months after recovering from SARS-CoV-2 infection for evaluation of cognitive difficulties. In addition to endorsing dysgeusia and dysnomia, she reported a variety of psychiatric symptoms and behavioral changes, including persistent anxiety and depression, apathy, and irritability features that she did not experience pre-COVID-19. She also self-reported ‘brain fogginess' described as difficulty concentrating (including on her schoolwork) and delayed thought process during the entire course of her illness with COVID-19. During a follow-up visit nine months after initial infection, the patient continued to struggle with word-finding difficulties and engaging in planning tasks or multitasking. She reported fatigue and apathy but noted that some of her symptoms, including her depression, had improved. She denied any history of seizures, stroke, anoxia injuries, or surgical intervention to the brain for any reason. The patient also denied having motor symptoms, gait disturbance, lateralized sensory/motor weakness, vision difficulty, auditory or visual hallucinations, obstructive sleep apnea, or REM sleep disinhibition. In terms of sleep health, she indicated that she averages 10 hours of sleep per night. She denied any pain but reported that her migraine frequency is approximately two migraines per week. She also endorsed a history of four reported concussions, one of which included a loss of consciousness at the age of 12.

### 2.2. Case #2

A 56-year-old South Asian-American female with a history of hypertension, type 2 diabetes, hypothyroidism, mixed connective tissue disorder, and lupus on Plaquenil presented to the Memory Disorders Clinic with language and memory difficulties after developing COVID-19 syndrome with a positive SARS-CoV-2 RT-PCR result one month earlier. She reported progressively worsening memory loss since five years prior that was noted to worsen after recovering from her recent SARS-CoV-2 infection. The patient replaced common words with sound-alike words and endorsed difficulty searching for the correct terminology. Additionally, she reported difficulty in recalling conversations and losing her train of thought when discussing topics with other individuals. She often double checked that she closed the garage door or turned off the stove in the kitchen. Though she had not gotten lost while driving, she had incidents in which she was driving and did not know where she was or where she was headed. The patient works as a physician assistant in neurology; however, her language difficulties have not impaired her ability to function at work and interact with patients.

### 2.3. Case #3

A 50-year-old African-American female with a history of depression, hypertension, and migraines presented to the clinic with a chief complaint of inattention and difficulties in executive function after she tested positive for SARS-CoV-2 five months prior to her initial clinic visit. She described her memory difficulties as ‘drawing blanks', including entering a room and not recalling why she entered the room in the first place or recalling passwords at her workplace. Her family noted concerns that she was forgetting conversations and she self-reported misplacing various items and having to think about where those items might be located.

## 3. Diagnostic Results

### 3.1. Case #1

A Montreal Cognitive Assessment (MoCA) performed during the patient's initial visit to the Memory Disorders Clinic revealed a score of 28/30, with a score of 30/30 on a repeat testing four months later. Given mild executive dysfunction that did not impair her daily function, along with normal results on the MoCA, the patient was diagnosed with subjective cognitive impairment. Neuropsychological testing showed mild executive function difficulty with a low-average score on digit-symbol substitution, but other executive functions and overall neurocognitive function was considered normal. She endorsed mild depression and anxiety on self-report measures. Furthermore, examination of the brain MRI revealed normal findings without evidence of restriction diffusion, white matter or brain matter atrophy, and hyperintensity or hemorrhage. The patient's NeuroQuant Age-Related Atrophy Report showed a hippocampal occupancy in the 68^th^ percentile. Given limited data for age-matched controls, the NeuroQuant Triage Brain Atrophy Report was not available for this patient.

### 3.2. Case #2

**Figure 1:**
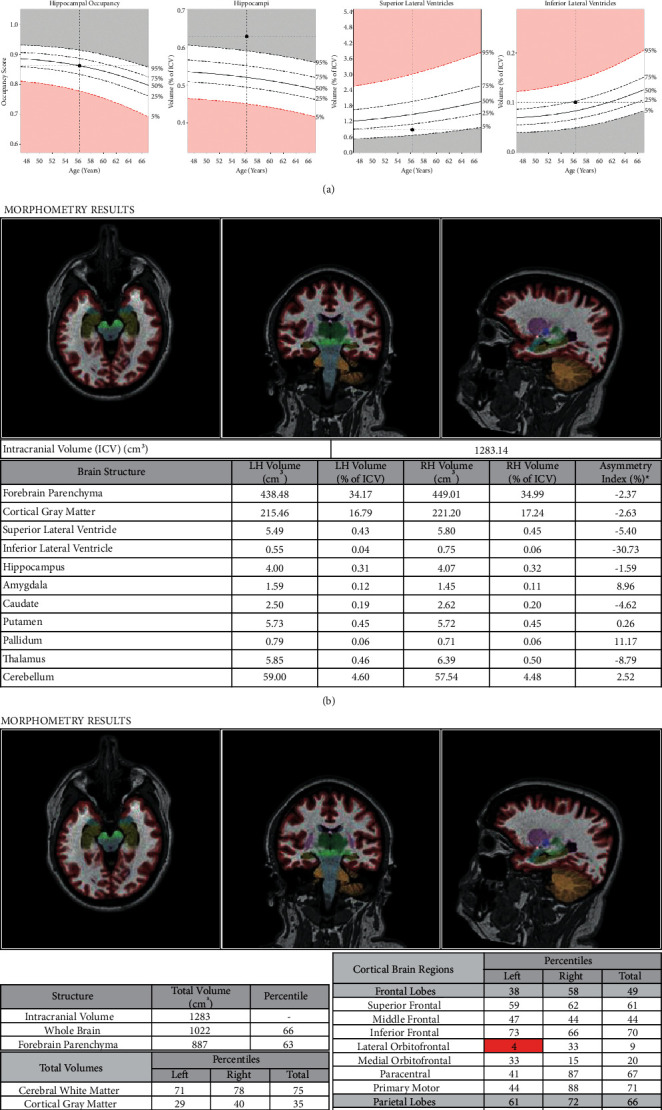
a-c. NeuroQuant report for case #2.

The patient scored a 29/30 on the MoCA during her visit, and given reported inattention and executive dysfunction that did not impair her daily function with a normal range on neuropsychological screening, she was diagnosed with subjective cognitive impairment. Results from formal neuropsychological testing performed one month after the initial clinic visit showed overall normal cognitive function except for mildly reduced processing speed of unclear clinical significance. There was no report of emotional distress on questionnaires. MRI of the brain performed three months after the clinic visit was normal without any evidence of restricted diffusion, hemorrhage, infarcts, or intracranial masses. Evaluation for hippocampal atrophy with NeuroQuant revealed a hippocampal volume in the 99^th^ percentile and a hippocampal occupancy score in the 51^st^ percentile. Further, atrophy was noted in the left lateral oribitofrontal as well as both left and right lateral occipital regions on the NeuroQuant Triage Brain Atrophy Report (Figure 1).

### 3.3. Case #3

The patient underwent a MoCA during her visit to the Memory Disorders Clinic and scored a 26/30. She was diagnosed with subjective cognitive impairment as daily function was not impaired by her executive function, memory, and inattention symptoms. Neuropsychological testing completed a month later revealed reassuring results with the exception of difficulties in verbal memory. She endorsed mild depression and anxiety on self-report measures. An MRI of the brain a month after the initial visit was normal. NeuroQuant analysis revealed a hippocampal volume in the 63^rd^ percentile and a hippocampal occupancy score in the 70^th^ percentile, along with evidence of atrophy in the left posterior superior temporal sulcus, left entorhinal cortex, and the left and right lateral occipital regions of the brain.

## 4. Discussion

In this case report, we describe three patients under the age of 60 who presented to the Memory Disorders Clinic at Duke University with complaints of cognitive dysfunction after recovery from SARS-CoV-2 infection. We sought to address several key questions, including the collection of neurologic and cognitive PASC symptoms, how long symptoms potentially last in this patient population, and whether any objective findings could be identified on imaging or neuropsychological testing. The patients, all of whom were diagnosed with subjective cognitive impairment, noted improvements in their cognition at the time of the clinic visit or on follow-up visits, and two were able to return to work after a period of leave. Assessment of their cognitive function, either with a MoCA screening tool or follow-up neuropsychological testing, revealed reassuring results (Table 1). Overall, these findings from neuropsychological testing suggest that in a young cohort of patients who develop cognitive sequelae from SARS-CoV-2 infection, their symptoms continue to improve with time. Analysis of the brain MRI of each of the three patients in this case report revealed normal hippocampal volume adjusted to age-related controls. Case #2 showed evidence of atrophy in the orbitofrontal cortex, which may be suggestive of executive dysfunction, though executive function was largely normal on neuropsychological testing. Further, cases #2 and #3 revealed atrophy in the occipital regions of the cortex, although this is of unclear significance. Longitudinal assessment with ongoing follow-up neuropsychological testing and brain imaging will better reveal the long-term clinical trajectory of these cognitive symptoms. This will also help determine if these patients are at risk for the development of a neurodegenerative disorder later in life.

Several reports have previously outlined the various symptoms that persist months after acute SARS-CoV-2 infection. The most common PASC symptoms that were noted two to six months post-infection included fatigue, dyspnea, anosmia/ageusia, anxiety, and depression [12, 18, 19], with studies suggesting more frequent incidence of PASC symptoms in those with severe disease hospitalized for COVID-19 [19]. Common neurological manifestations that have been reported in long-haulers include sleep disturbances, headaches, fatigue, cognitive impairment, and difficulties in sustaining attention [20–22]. While these studies examined the collection of PASC symptoms after initial infection in large cohorts of patients, no studies have yet stratified the symptomatology between younger and older cohorts.

The National Institute for Health and Care Excellence (NICE) in the U.K. has published guidelines to assess the clinical features of the long-term consequences of SARS-CoV-2 infection. NICE defines three stages reflecting symptomatic recovery, including the following: (1) acute COVID-19, which refers to symptoms up to 4 weeks or 28 days after the onset of infection with SARS-CoV-2; (2) ongoing symptomatic COVID-19, generally defined as symptoms ranging from four to 12 weeks after onset of infection; and (3) post-COVID-19, referring to symptoms associated with SARS-CoV-2 infection that continue for 12 weeks or greater [23]. Symptoms persisting beyond the acute phase of SARS-CoV-2 infection are commonly referred to as “long-COVID”, while a “long hauler” refers to a patient who has recovered from acute SARS-CoV-2 infection experiencing persistent symptoms [23, 24]. In February 2021, the National Institutes of Health in the U.S. defined the collection of symptoms associated with long-COVID as PASC to identify the potential causes, treatment and prevention of those suffering from these long-term effects of COVID-19. However, the broad clinical phenotype and unknown duration of PASC requires further investigation to comprehensively characterize this syndrome.

With an increase in the number of cases and hospitalization rates due to the surge in variants, there continues to be an increasing number of patients who develop persistent symptoms beyond the acute phase of infection. Several crucial questions such as follows remain: (1) Does the severity of cognitive dysfunction secondary to SARS-CoV-2 correlate with severity of disease in young cohorts? Younger patients generally tend to have fewer medical comorbidities and are at reduced risk for more severe infection compared to the older population. Certainly, PASC symptoms have been identified in both patients with mild disease requiring outpatient treatment as well as in those hospitalized with more severe illness, but risk has been noted to be greater in those with severe COVID-19 [20]. (2) Are females at increased risk of developing cognitive dysfunction after recovery from SARS-CoV-2 infection compared to males? As findings from recent studies suggest a predominance of PASC in females [21], it will be crucial to determine whether cognitive dysfunction also has a female : male predominance, and what the contributing risk factors are. (3) Does the landscape of cognitive symptoms differ in younger *versus* older patients, and is the timeline of these symptoms different between these two populations? Further, are there any significant changes on brain MRI that occur longitudinally after recovery from SARS-CoV-2 infection? (4) Given concerns over emerging COVID-19 variants, do the characteristics of cognitive symptoms depend on the specific variant? (5) Finally, what role does vaccination play in mitigating the effects in both who previously developed and recovered from COVID-19 illness and those who have not been infected with SARS-CoV-2 but are still at risk? While these questions are beyond the scope of our study, there is a dire need to elucidate the pathophysiology and identify the risk factors and treatment modalities for those suffering from persistent symptoms secondary to SARS-CoV-2.

## 5. Methods

Clinical neuropsychological evaluations of these individuals included a fixed flexible battery. Neuropsychological tests were administered in standard fashion according to test manuals. Publisher information and additional details about these tests can be found elsewhere [25]. A master's-level psychometrist administered the neuropsychological tests under the supervision of a clinical neuropsychologist. The neuropsychological test results in Table 1 include raw scores, standard scores (SS), and T-scores, as well as raw scores and T-scores where applicable. A standard score has a mean of 100 and a standard deviation of 15. A *T* score has a mean of 50 and a standard deviation of 10. It is to be noted that because this was a fixed flexible battery there are times in which certain subtests were not administered due to a patient's tolerance for testing or due to difficulties with psychometric properties of the test. If a test was not administered, it was indicated on the table with NA.

Test measures included the following:

The Test of Premorbid Functioning (TOPF) is an assessment tool to estimate premorbid cognitive ability that requires an examinee to read a list of phonetically irregular words. The TOPF provides multiple possible scores, including a predicted score and achieved score. Table 1 includes an actual achieved score.

Wechsler Adult Intelligence Scale - Fourth Edition (WAIS-IV) is a measure of intellectual function used to derive a full-scale IQ score and four indices based on verbal comprehension, perceptual reasoning, working memory, and processing speed.

Trail Making Test (A&B) : Part A of this test assesses simple visual sequencing, psychomotor speed, and general processing speed by requiring the examinee to quickly draw lines sequentially in a numerical order from 1–25. Part B of the test is similar but requires alphanumeric sequencing (e.g., 1, A, 2, B, 3, C, etc.). Part B is considered more complex and measures cognitive flexibility. These tests are sensitive to neurological impairment and are often categorized as measures of executive function. The raw score on Table 1 is the total time to completion.

Wisconsin Card Sorting Test (WCST) : This test assesses an individual's strategic planning and ability to utilize environmental feedback and problem solve. It is a common measure of executive function. It is sensitive to frontal lobe damage, particularly related to dorsolateral prefrontal regions. There are multiple subscores that can be derived from this test but the table presents the number of categories generated out of 6 possible categories.

California Verbal Learning Test - Second Edition (CVLT-II) : As a test of verbal episodic memory, this test assesses for verbal learning and recall by requiring an examinee to repeat back a list of words over multiple trials and to recall as many words as possible after a delay.

Brief Visuospatial Memory Test - Revised (BVMT-R) : As a visual memory test assessing episodic memory, this test requires an examinee to recall shapes and to draw them in the same location presented on a page. This test includes learning trials, a delayed recall trial, and a recognition memory trial.

Boston Naming Test (BNT) : This is a common test of confrontational object naming in which an examinee is presented with simple line drawings and asked to name the item. Phonemic and semantic cues are often provided for individuals who cannot identify the object. Table 1 includes a raw score out of 60 items.

Controlled Oral Word Associations Test (FAS) and Category Naming (Animal Fluency): Verbal fluency measures required individuals to rapidly name as many objects as they can that either begin with a certain letter or fall under a certain semantic category. These are one minute trials. Phonemic fluency is mediated by frontal regions, whereas semantic fluency is mediated by left temporal and inferior parietal regions.

Beck Depression Inventory - Second Edition (BDI-II) and Beck Anxiety Inventory (BAI) : These self-report questionnaires assess for symptoms of depression occurring over the most recent 2 weeks and symptoms of anxiety occurring over the most recent week.

## Figures and Tables

**Table 1:** Neuropsychological findings in the three cases after recovery from SARS-CoV-2 infection.

Demographics	Case 1	Case 2	Case 3
Age	56	19	49
Sex	Female	Female	Female
Education	18	13	18
Race	Asian	W/non-Hisp	Black
Tests			
TOPF (Actual)	107	111	90
Intellectual Functioning			
WAIS-IV			
FSIQ (SS)	103	123	NA
GAI (SS)	102	132	101
VCI (SS)	100	143	98
PRI (SS)	105	115	104
WMI (SS)	114	111	NA
PSI (SS)	92	100	NA
Executive Functioning			
TMT A (Raw) T	21 (58)	17 (59)	26 (49)
TMT B (Raw) T	50 (53)	36 (59)	74 (47)
WCST Categories (Raw)	6	6	6
Digit Span	34 (63)	30 (53)	31 (57)
Coding	53 (47)	57 (40)	70 (53)
Language			
BNT (Raw) T	44 (21)	57 (53)	52 (41)
FAS (Raw) T	39 (43)	33 (39)	60 (64)
Animal (Raw) T	19 (43)	23 (52)	25 (60)
Vocabulary (Raw) T	37 (50)	49 (70)	43 (53)
Similarities (Raw) T	29 (57)	35 (80)	30 (57)
Verbal Memory			
CVLT-II			
Total Learning (Raw) T	60 (63)	63 (60)	37 (36)
Delayed Recall (Raw) T	13 (55)	14 (55)	6 (30)
Visual Memory			
BVMT-R			
Total Learning (Raw) T	NA	29 (61)	25 (52)
Delayed Recall (Raw) T	NA	12 (66)	12 (65)
Mood			
BDI-II	7	17	18
BAI	8	8	13
NA = Not administered			
